# Reassessing the role of external fixation in polytrauma and severly injured patients with chest trauma and femur fracture: a propensity score-matched analysis from the TraumaRegister DGU^®^

**DOI:** 10.1007/s00068-025-02956-x

**Published:** 2025-09-23

**Authors:** Annette Keß, Christian Kleber, Rolf Lefering, Georg Osterhoff

**Affiliations:** 1 Department for Orthopedics, Trauma and Plastic Surgery, University Hospital Leipzig, 04103 Leipzig, Germany; 2 Institute for Research in Operative Medicine, University Witten/Herdecke, 51109 Cologne, Germany

**Keywords:** Femoral fractures, Thoracic injuries, Intramedullary nailing, External fixation, Critical care

## Abstract

**Introduction:**

Early intramedullary nailing for femur fractures in patients with thoracic trauma has been controversial due to concerns about exacerbating pulmonary complications, particularly in cases of lung contusion. Older studies, relying on conventional radiographs that detected only severe contusions, recommended damage control surgery with external fixation. Today, high-resolution computed tomography (CT) detects even mild lung contusions, potentially overestimating lung damage. Coupled with modern ventilation strategies, this has prompted a reassessment of early definitive fixation versus external fixation in these patients.

**Methods:**

Data from 3,087 patients with femur fractures and chest trauma (Abbreviated Injury Scale (AIS) ≥ 2) were extracted from the TraumaRegister DGU^®^ between 2019 and 2023. A propensity score-matched analysis (*n* = 2,014) was conducted to compare patients treated with intramedullary nailing (IMN) and external fixation. Clinical endpoints included mortality, Intensive Care Unit (ICU) and hospital length of stay, multiple organ failure, and blood transfusion rates.

**Results:**

Approximately half of the patients (46.8%) received initial external fixation of the femur fracture. External fixation was associated with higher injury severity score (ISS 31.5 vs. 26.1; *p* < 0.001) and were more frequently treated in Level-1 trauma centers and during night shifts. A propensity score matched analysis revealed no significant differences in mortality (6.0% early definitive care vs. 7.4% external fixation; *p* = 0.212) or multiple organ failure rates (25.3% vs. 29.4%; *p* = 0.091). However, external fixation patients had longer ICU stays (median 10 vs. 7 days; *p* < 0.001) and hospital stays (median 25 vs. 20 days; *p* < 0.001).

**Conclusion:**

These findings suggest that external fixation of femur fractures may not be universally necessary, particularly in patients with moderate chest trauma, and early definitive care may offer comparable outcomes with shorter recovery times. Standardized criteria are needed to optimize treatment decisions and reserve external fixation for cases where it is clinically essential.

## Introduction

Early fixation of femur fractures is known to reduce the risk of complications associated with extended periods of immobilization. However, in patients with concomitant thoracic trauma, early femur fixation by intramedullary nailing has been historically controversial due to concerns that it could exacerbate pulmonary injury [1, 2]. Studies, including those by Pape et al. [3], have suggested that early femur fracture fixation in patients with thoracic trauma may increase the risk of complications, such as respiratory distress and acute respiratory distress syndrome (ARDS) particularly when lung contusion was diagnosed [4]. Hence, it was recommended for these patients to proceed with a damage control surgery (DCS) approach, initially with external fixation and subsequently with definitive treatment in a two-stage process.

Most of these studies were based on conventional radiographs that often failed to identify milder or more moderate lung contusions, potentially leading to a delayed understanding of the severity of the thoracic injury and influencing the treatment decisions for femur fractures.

In recent years, however, significant advances in trauma care have changed the landscape of managing patients with both femur fractures and thoracic trauma. The widespread use of high-resolution computed tomography (CT) in the trauma bay has dramatically improved the detection of even subtle lung contusions, offering a more detailed and early understanding of the extent of pulmonary injury [5]. Additionally, modern anesthetic and ventilation techniques, such as protective lung ventilation strategies, have proven effective in mitigating the risk of ARDS, even in patients with significant thoracic injury. These advancements have made it possible to reassess the traditional concerns about early surgical intervention for femur fractures in the context of thoracic trauma [6].

The aim of this study is to evaluate the impact of early femur intramedullary nailing (IMN) in polytrauma and severly injured patients with thoracic trauma of varying severity. Our central hypothesis posits that the widespread adoption of CT imaging has increased the detection of even mild lung contusions. This, in turn, has led to the potential “overdiagnosis” of severe thoracic trauma and to an altered the risk profile for patients with thoracic trauma. By detecting less severe injuries early, clinicians are now better equipped to manage these conditions with reduced concern about exacerbating pulmonary complications through early surgical fixation of femur fractures.

We further hypothesize that, given these advancements, many patients with moderate thoracic injury who were traditionally managed with DCS and delayed femur fracture fixation could now benefit from early definitive treatment. This study seeks to reassess the role of DCS in the management of femur fractures in polytrauma and severly injured patients with thoracic trauma, exploring whether the current diagnostic and therapeutic advancements have shifted the balance in favor of earlier intervention, leading to improved patient outcomes. Through a comprehensive analysis of current outcomes, we seek to refine the management protocols for polytrauma and severly injured patients, optimizing fracture care while minimizing the risk of pulmonary complications.

## Methods

### TraumaRegister DGU®

The TraumaRegister DGU^®^ of the German Trauma Society (Deutsche Gesellschaft für Unfallchirurgie, DGU) was founded in 1993. The aim of this multi-centre database is a pseudonymised and standardised documentation of severely injured patients.

Data are collected prospectively in four consecutive time phases from the site of the accident until discharge from hospital: (A) pre-hospital phase, (B) emergency room and initial surgery, (C) intensive care unit and (D) discharge. The documentation includes detailed information on demographics, injury pattern, comorbidities, pre- and in-hospital management, course on intensive care unit, relevant laboratory findings including data on transfusion and outcome of each individual. The inclusion criteria are admission to hospital via emergency room with subsequent ICU/ICM care, or arrival at the hospital with vital signs but death occurring before admission to the ICU.

The infrastructure for documentation, data management, and data analysis is provided by AUC - Academy for Trauma Surgery (AUC - Akademie der Unfallchirurgie GmbH), a company affiliated to the German Trauma Society. The scientific leadership is provided by the Committee on Emergency Medicine, Intensive Care and Trauma Management (Sektion NIS) of the German Trauma Society. The participating hospitals submit their pseudonymised data to a central database through a web-based application. Scientific data analysis is approved according to a peer review procedure laid down in the publication guideline of TraumaRegister DGU^®^.

The present study is in line with the publication guidelines of the TraumaRegister DGU^®^ and registered as TR-DGU project ID 2024-001.

The participating hospitals are primarily located in Germany (90%), but a rising number of hospitals of other countries contribute data as well (at the moment from Austria, Belgium, China, Finland, Luxembourg, Slovenia, Switzerland, The Netherlands, and the United Arab Emirates). Currently, more than 38,000 cases from almost 700 hospitals are entered into the database per year. Participation in TraumaRegister DGU^®^ is voluntary. For hospitals associated with TraumaNetzwerk DGU^®^, however, the entry of at least a basic data set is obligatory for reasons of quality assurance.

### Study population

Between 2019 and 2023, data from all patients with femur fractures and chest trauma were extracted from the TraumaRegister DGU^®^ (TR-DGU). Inclusion criteria were chest trauma with Abbreviated Injury Scale (AIS) ≥ 2, data from European hospitals, patient age ≥ 16 years, with surgical treatment of the femur fracture. According to the AIS, a score of ≥ 2 encompasses thoracic injuries starting from the fracture of two or more ribs, unilateral pulmonary contusions or parenchymal injuries affecting less than one lobe, as well as pneumothorax. Patients who died within the first 24 h, or were transferred in from another hospital, or were transferred out within 48 h (no outcome available) were excluded from the analysis. We divided the study population into two groups, the EDC-group, which was treated primary with INM, and the DCS-group, which was treated primary with an external fixation.

### Statistical analysis

Descriptive multivariate analysis bad been performed using SPSS statistical software (version 29; IBM Inc., Armonk, NY, USA). Categorical data were presented as numbers with percentage, and metric variables were presented as mean with standard deviation, or median with quartiles, depending on the distribution. Comparisons were performed with the chi-squared test for categories, and Mann-Whitney U-test for metric data, respectively. A p-value < 0.05 was considered statistically significant.

A multivariate logistic regression analysis with external fixation as dependent variable was used to create a propensity score. The following variables were included: age, sex, penetrating trauma, hospital level of care (1,2,3), admission at nighttime, admission on weekends, blood transfusion in the emergency room/operating room ER/OR, severity of chest trauma (AIS: 2/3/4/5), Polytrauma (Berlin definition) [7], Shock: systolic blood pressure ≤ 90 mmHg (preclinical or on admission), unconsciousness (GCS 3–8), coagulopathy, injury pattern (body regions head, abdomen, spine, and p elvis, each with AIS ≥ 2). Non-relevant predictors (*p* > 0.10) were deleted from the final propensity score.

The parameters acidosis and hypothermia could not be included in the analysis, as the data are either not provided in the TraumaRegister DGU or inconsistently documented.

Based on the propensity score (rounded values as percentage) which is the probability of receiving an external fixation of the femur, we were able to match 1007 patients with definitive care and 1007 patients with external fixation. Matching was performed randomly and blinded to outcome, with a ± 1% tolerance. The clinical endpoints were mortality, risk of death (based on RISC II), hospital mortality, length of stay (ICU), length of stay (hospital), and multiple organ failure and blood transfusion.

The Revised Injury Severity Classification (RISC) score version II provides a risk of death prediction; it has been developed and repeatedly validated using data from the TR-DGU [8].

## Results

**Fig. 1:**
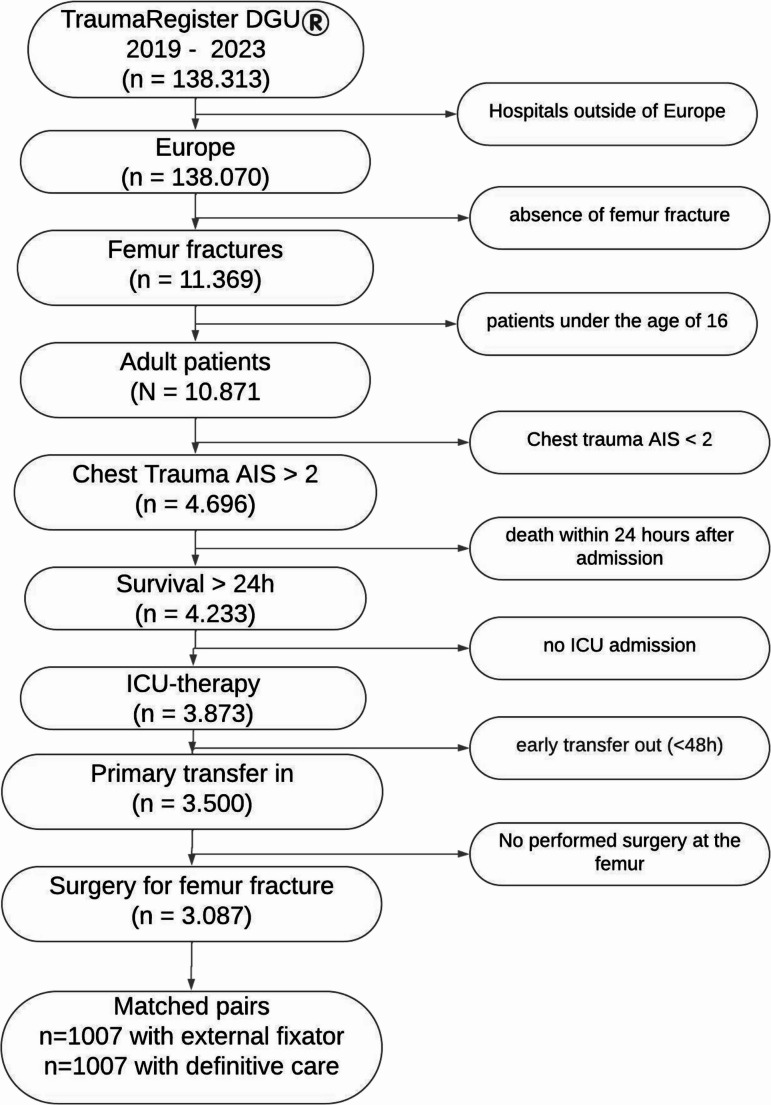
Patient selection flow chart

A total of 3,087 patients with chest trauma (AIS ≥ 2) and surgery after femur fracture from 437 European hospitals were included. The mean age was 48 years, and 73.4% were males. The mean Injury Severity Score (ISS) was 28.6 points, and the mortality rate was 7.6% (deaths < 24 h were excluded) (Fig. 1).

**Fig. 2:**
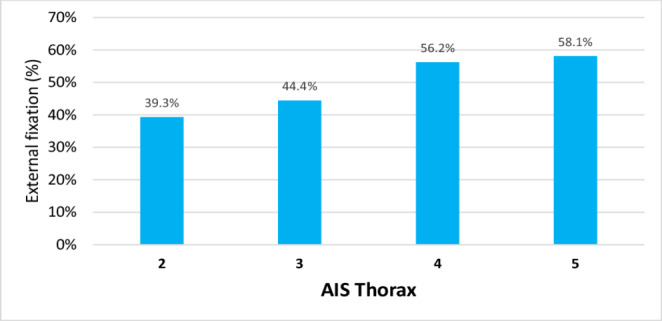
Percentage of patients treated with external fixation by AIS Thorax (unadjusted data)

**Fig. 3:**
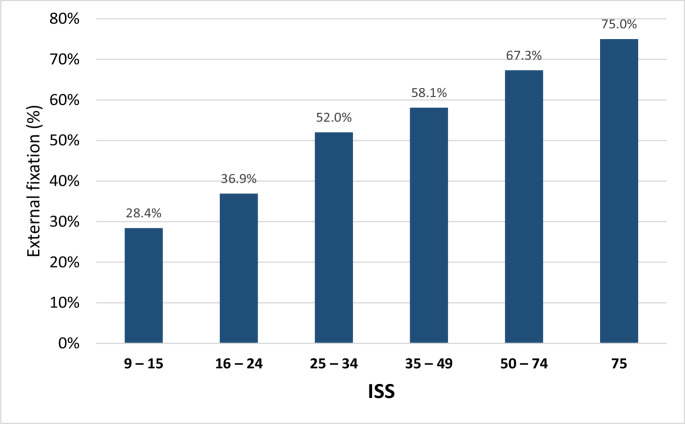
Percentage of patients treated with external fixation by ISS (unadjusted data)

**Fig. 4:**
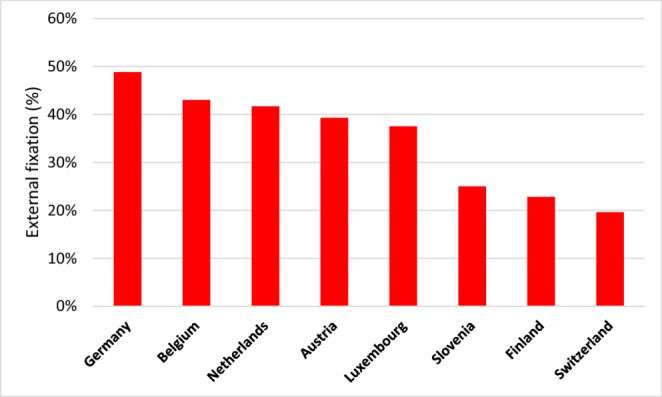
Percentage of patients treated with external fixation by country (unadjusted data)

Approximately half of the patients (46.8%) were initially treated in terms of DCS with initial external fixation. Regarding the unadjusted analysis, a higher AIS chest (*p* = 0.364) and a higher total ISS (*p* < 0.001) were associated with a higher likelihood for external fixation as primary treatment of femur fractures (Figs. 2 and 3). Level-1 trauma centers (52.6%, Level-2: 31.9%, Level-3: 19.2%, *p* < 0.001) and trauma centers in Germany (48.8%, *p* < 0.001, Fig. 4) were more likely to use external fixation. In addition, external fixation was applied more frequently during nightshifts (from 6pm to 6am, 51.8%) compared to daytime admissions (44.0%, *p* < 0.001). In contrast, increasing age was associated with a higher rate of primary intramedullary nailing (*p* < 0.001) (Table 1).

**Table 1:** Demographic and prehospital characteristics of all patients and of propensity score matched pairs

	All Patients			Matched pairs		
	EDC (*n* = 1641)	ExFix (*n* = 1446)	*p*-value	EDC (*n* = 1007)	ExFix (*n* = 1007)	*p*-value
Age [years]	52.3 (21.4)	43.1 (19.1)	<.001	46.8 (18.8)	44.3 (20.0)	.002
Age >60 years	631 (38.5%)	327 (22.6%)	<.001	261 (25.9%)	268 (26.6%)	.723
Sex [N male]	1190 (72.5%)	1076 (74.4%)	.237	776 (77.1%)	740 (73.5%)	.071
Level 1 Hospitals	1040 (63.4%)	1197 (82.8%)	<.001	768 (76.3%)	790 (79.6%)	.241
German Hospitals	1337 (81.5%)	1275 (88.2%)	<.001	785 (78.0%)	902 (89.6%)	<.001
ISS	26.1 (10.4)	31.5 (12.4)	<.001	28.5 (11.3)	29.1 (11.3)	.231
Penetrating injury	8 (0.5%)	32 (2.2%)	<.001	8 (0.8%)	5 (0.5%)	.580
GCS 3 - 8	163 (9.9%)	324 (22.4%)	<.001	151 (15.0%)	158 (15.7%)	.711
AIS head 2+	478 (29.1%)	604 (41.8%)	<.001	356 (35.4%)	348 (34.6%)	.744
Weekend admission	708 (43.1%)	611 (42.3%)	.636	449 (44.6%)	422 (41.9%)	.242
Nightshift admission	543 (33.1%)	584 (40.4%)	<.001	376 (37.3%)	381 (37.8%)	.854
AIS-Chest	381 (23.2%) 892 (54.4%) 233 (14.2%) 135 (8.2%)	247 (17.1%) 713 (49.3%) 299 (20.7%) 187 (12.9%)	.364	216 (21.4%) 512 (50.8%) 169 (16.8%) 110 (10.9%)	193 (19.2%) 528 (52.4%) 175 (17.4%) 111 (11.0%)	.364
Polytrauma (Berlin Definition)	773 (47.1%)	861 (59.5%)	<.001	485 (48.2%)	509 (50.2%)	.373
Coagulopathy	219 (13.8%)	346 (24.7%)	<.001	159 (15.8%)	183 (18.2%)	.171
Blood Transfusion	363 (22.1%)	628 (43.4%)	<.001	312 (31.0%)	323 (32.1%)	.632
Shock (BP<=90)	300 (18.3%)	503 (34.8%)	<.001	237 (23.5%)	269 (26.7%)	.111
ASA 3/4	333 (20.3%)	156 (10.0%)	<.001	144 (14.3%)	117 (11.6%)	.084

*EDC* Early definitive care, *ExFix* External fixation, *BP *Blood pressure, *AIS* Abbreviated Injury Scale

*ISS* Injury Severity Score, *GCS* Glasgow Coma Scale, *ASA* ASA Physical Status Classification System

Continuous data is reported as “mean (standard deviation)”, nominal data is reported as “frequency (percentage)”

In all cases, patients with initial external fixation were more severely injured (ISS 31.5 vs. 26.1; *p* < 0.001) and had more life-threatening conditions like severe head injury, being in hemodynamic shock, rate of blood transfusion, or coagulopathy (*p* < 0.001). However, compared to the Revised Injury Severity Classificatio, Version II (RISC-II) [8]prognosis, they had a better survival than expected. The in-hospital mortality in the external fixation group was 8.5% whereas the calculated risk of death (based on RISC II) was 12.5%. In the early definitive care group the calculated risk of death (based on RISC II) was 7.2% whereas the in-hospital mortality was 6.8%. However, this study excluded patients who died within 24 h which likely causes a relevant bias.

A propensity score (i.e. the probability to receive primary external fixation) was calculated using a multivariable logistic model. Penetrating trauma, younger age (< 60 years), hypovolemia (blood transfusion and shock), and level-1 trauma center emerged as the strongest predictors for the use of external fixation (Table 2).

**Table 2:** Outcome of all patients and of propensity score matched pairs

	All Patients			Matched pairs		
	EDC (*n* = 1641)	ExFix (*n* = 1446)	p-value	EDC (*n* = 1007)	ExFix (*n* = 1007)	*p*-value
Length of stay ICU [days]	5 (2–13)	12 (5–23)	<.001	7 (3–16)	10 (4–20)	<.001
Length of ventilation [days]	1 (0–4)	4 (1–13)	<.001	1 (0–7)	2 (0–10)	<.001
Length of stay hospital [days]	18 (12–29)	28 (18–43)	<.001	20 (12–32)	25 (17–40)	<.001
Hospital mortality	112 (6.8%)	123 (8.5%)	.089	60 (6.0%)	75 (7.4%)	.212
Risk of death (based on RISC II)	7.2%	12.5%	.135	8.0%	9.3%	.135
Multiple Organ Failure (MOF)	198 (21.4%)	401 (35.7%)	<.001	161 (25.3%)	218 (29.4%)	.091

*EDC* Early definitive care, *ExFix* External fixation, *ICU* Intensive care unit, *RISC II* Revised Injury Severity Classification, 2^nd^ edition. Continuous data is reported as “mean (standard deviation)”, nominal data is reported as “frequency (percentage)”

A total of 1,007 pairs (2,014 patients) were successfully matched, including 993 pairs with identical propensity scores and 14 pairs with a +/- 1% deviation. This corresponds to 65.2% of patients with a femur fracture being included in the matching process. A total of 103 cases were excluded due to missing data.

The two cohorts were well-matched in terms of demographic characteristics, injury patterns, and severity of chest trauma (Table 1).

The mean ISS was 28.5 in the early definitive care group and 29.1 in the external fixation group (*p* = 0.231). Half of the patients in each group had a chest trauma with AIS = 3 (50.8% early definitive care, 52.4% external fixation, *p* = 0.364) and 11% in each group had a chest trauma AIS = 5. Patients in German hospitals were significantly more likely to receive external fixation compared to patients in European centers outside Germany participating in the registry (48.8% vs. 36.04%, *p* < 0.001).

Half of the patients in both groups were polytraumatized patients according to the Berlin definition. About one quarter of the patients (23.5% early definitive care vs. 26.7% external fixation, *p* = 0.111) presented with hemodynamic shock and a third received blood transfusion (31.0% early definitive care vs. 32.1% external fixation; *p* = 0.632) (Table 1).

Regarding outcomes, no significant differences were observed between the two matched groups: The in-hospital mortality was 6.0% for the early definitive care group compared to 7.4% in the external fixation group (*p* = 0.212), while the calculated risk of death based on the RISC II score was 8.0% for the early definitive care group and 9.3% for the external fixation group (*p* = 0.135). Regarding intrahospital morbidity, 25.3% of patients in the early definitive care group and 29.4% in the external fixation group (*p* = 0.091) developed multiple organ failure. Although there were no significant differences in hospital mortality between the two groups, patients treated with external fixation had a significantly longer intensive care unit stay (median 10 vs. 7 days, *p* < 0.001) and total hospital stay (median 25 vs. 20 days, *p* < 0.001) (Table 3).

**Table 3:** Logistic regression model with external fixator as surgical treatment as dependent variable. The probability for receiving an external fixator served as propensity score for the subsequent matching

	Regression coefficient	Odds Ratio	*p*-value
Age [years] (ref: <60 years)			
60–69	−0.259	0.77	0.027
70–79	−0.832	0.44	< 0.001
> 80	−1.429	0.24	< 0.001
Penetrating Trauma (ref: blunt)	1.349	3.85	0.002
Level of Trauma Center (ref: 1)			
2	-0.578	0.56	< 0.001
3	−0.888	0.41	< 0.001
Nightshift Admission	0.288	1.33	< 0.001
Blood Transfusion	0.539	1.71	< 0.001
AIS-Chest (ref: 2)			
3	0.121	1.13	0.265
4	0.270	1.31	0.050
5	0.412	1.51	0.010
Shock (BP < = 90)	0.288	1.33	0.015
Coagulopathy	0.243	1.275	0.036
GCS 3–8	0.294	1.34	0.022
Polytrauma (Berlin Definition)	0.207	1.23	0.065
AIS Head > 2	0.218	1.24	0.015
AIS Abdomen > 2	0.227	1.26	0.011

*BP* Blood pressure, *AIS* Abbreviated Injury Scale, *GCS* Glasgow Coma Scale, *Ref *Reference category

## Discussion

This study evaluated the outcomes and characteristics of 3,087 patients extracted from the TraumaRegister DGU^®^ with chest trauma and femur fractures from European hospitals, comparing those treated with primary external fixation in terms of DCS to those receiving early definitive care. The findings revealed several important patterns regarding treatment choices, patient characteristics, and clinical outcomes, with implications for future clinical decision-making and trauma care strategies.

Our analysis showed that nearly half of the patients were initially treated with external fixation, and this treatment modality was more frequently employed in patients with higher injury severity, particularly those with an increased AIS for chest trauma and higher ISS. This is consistent with previous studies indicating that more severe trauma is associated with a greater likelihood of external fixation, particularly in polytrauma patients [9]. The use of external fixation was more common in Level-1 trauma centers. These results suggest that more specialized centers tend to opt for external fixation, likely because managing severe fractures in the context of polytrauma requires more advanced care capabilities. Furthermore, patients treated at Level-1 trauma centers are more likely to present with severe injuries involving multiple body regions, not limited to the chest and femur [10]. 

A noteworthy finding was the increased frequency of external fixation application during night shifts compared to daytime. This could reflect both the time-sensitive nature of trauma management during off-hours and possibly differences in staff availability or decision-making processes. A recent study from the TraumaRegister DGU^®^ covering the years 2007 to 2017 showed that trauma patients admitted during nighttime are younger, more severely injured (as indicated by a higher Injury Severity Score, ISS), and present with lower Glasgow Coma Scale (GCS) scores, an indicator of head trauma. These factors are relevant for the decision to perform DCS and may also explain the higher rates of external fixation during night shifts [11]. 

Although these observations warrant further investigation, they do raise interesting questions about how trauma care practices might vary by time of day.

In the unadjusted analysis, we found that patients treated with external fixation were more severely injured, as evidenced by higher ISS scores (31.5 vs. 26.1), and had a higher incidence of associated complications such as severe head injuries, shock, and coagulopathy. Despite these greater challenges, the mortality rate in the external fixation group (8.5%) was lower than the predicted mortality rate based on the RISC-II prognosis (12.5%). This suggests that patients who received external fixation may benefit from this treatment approach in terms of survival, potentially due to more effective management of their fractures and associated injuries, although the exact mechanisms remain to be further explored [12].

The propensity score matching analysis [13], which matched 2,014 patient pairs based on various predictors such as penetrating trauma, younger age, and trauma center level, revealed that the two groups were well-matched in terms of demographic data, injury patterns, and chest trauma severity. This strengthens the validity of our findings by reducing confounding biases. The matched cohorts exhibited similar morbidity levels, with around a tenth of both groups being classified as ASA 3 or 4, reflecting the significant degree of physiological stress these patients experienced [2].

Regarding clinical outcomes, there were no significant differences in in-hospital mortality between the two groups which is in line with the results of a recent meta-analysis [14]. The mortality rates were comparable, which suggests that the choice of fracture fixation method did not directly influence survival rates. However, patients in the external fixation group had a significantly longer length of stay in the intensive care unit and a longer total hospital stay. Recknagel et al. demonstrated in a rat model that the conversion from external fixation to IMN induces a second immunological hit, characterized by increased serum levels of pro-inflammatory cytokines and complement activation products—particularly when combined with chest trauma—which may contribute to a prolonged stay in the intensive care unit [15]. However, further studies in humans investigating this immunological second hit in the context of femur fractures and chest trauma are lacking.

The similar rate of multiple organ failure and blood transfusion suggests that the overall burden of acute physiological stress was comparable, despite the differences in fracture treatment. These findings likely reflect the increased complexity and severity of the injuries sustained by these patients, which required extended ICU care and prolonged hospitalization for stabilization and recovery.

In summary, the data show no significant differences in mortality or morbidity between the two groups, even among matched patients with similar injury patterns and chest trauma severity. This supports the hypothesis that external fixation may not be universally necessary, especially for patients with moderate chest trauma. The significantly longer ICU and hospital stays for the external fixation group suggest potential inefficiencies or complications associated with this approach. This finding aligns with our hypothesis by indicating that early definitive care might lead to faster recovery and discharge in some cases. Our observations are consistent with findings from several other studies [1, 16–19]. 

Given that patients treated with external fixation were more severely injured (higher ISS, shock, transfusion needs) but still achieved comparable survival rates, the necessity of external fixation in less critical cases could be questioned. This suggests a need to re-evaluate protocols for patients with severe chest trauma but without other life-threatening conditions. The higher rates of external fixation in specific contexts (e.g., German hospitals, night shifts) highlight the influence of institutional preferences rather than strictly clinical indications. In this context it is important to note that the vast majority of patients analysed in this study were treated in German hospitals. This supports the need for standardized criteria to guide treatment decisions, ensuring external fixation is reserved for cases where it is truly necessary. Among these guidelines are the recently published recommendations of the IMPACT group, which established 20 consensus statements regarding the timing of fracture fixation in patients with associated injuries.

However, with only three statements specifically addressing thoracic trauma and broadly formulated recommendations concerning major fractures, these guidelines remain limited in their applicability to the specific clinical scenario of femoral fractures in the presence of concomitant chest trauma [20]. 

Regarding the limitation of our study, we must consider the use of the propensity score. While propensity score methods offer a robust approach for reducing confounding in observational trauma research, several limitations must be considered when interpreting our findings. Propensity scores can only adjust for known and measured covariates; unmeasured confounders—such as the precise physiological response to combined thoracic and femoral trauma or variations in institutional treatment protocols—may still bias the results. Moreover, the validity of the matching process depends on the correct specification of relevant covariates, which is particularly challenging in heterogeneous trauma populations. In our study, cases without suitable matches were excluded, which may limit generalizability and reduce statistical power. Furthermore, although covariate balance was achieved in observed variables, residual confounding cannot be entirely ruled out, and causal inferences regarding the superiority of EDC or DCS in this subgroup should be made with caution.

## Conclusion

In conclusion, the data indicate no significant differences in mortality or morbidity between early definitive care and external fixation, even in matched patients with similar chest trauma severity, suggesting that external fixation may not be universally necessary, particularly in moderate cases. The longer ICU and hospital stay associated with external fixation highlight potential inefficiencies, reinforcing the need to re-evaluate protocols for patients without other life-threatening conditions. Standardized criteria should be developed to guide treatment decisions, ensuring external fixation is reserved for cases where it is clinically essential.

## Data Availability

No datasets were generated or analysed during the current study.

## References

[1] Jiang M, Li C, Yi C, Tang S. Early intramedullary nailing of femoral shaft fracture on outcomes in patients with severe chest injury: a meta-analysis. Sci Rep. 2016;6(1): 30566. 10.1038/srep30566. 27457468 10.1038/srep30566 PMC4960546

[2] Mert Ü, et al. Damage control orthopaedics induced less trauma-induced coagulopathy than early total care in a porcine polytrauma model. Eur Surg Res. 2024;65:115–22. 10.1159/000541399. 39348804 10.1159/000541399

[3] Pape HC, et al. Changes in the management of femoral shaft fractures in polytrauma patients: from early total care to damage control orthopedic surgery. J Trauma Inj Infect Crit Care. 2002;53(3):452–62. 10.1097/00005373-200209000-00010. 10.1097/00005373-200209000-00010 12352480

[4] Morshed S, Miclau T, Bembom O, Cohen M, Knudson MM, Colford JM. Delayed internal fixation of femoral shaft fracture reduces mortality among patients with multisystem trauma. J Bone Jt Surg -Am. 2009;91(1):3–13. 10.2106/JBJS.H.00338. 10.2106/JBJS.H.00338 PMC2663326 19122073

[5] Van Diepen MR, Wijffels MME, Verhofstad MHJ, Van Lieshout EMM. Classification methods of pulmonary contusion based on chest CT and the association with in-hospital outcomes: a systematic review of literature. Eur J Trauma Emerg Surg Off Publ Eur Trauma Soc. Sep. 2024. 10.1007/s00068-024-02666-w. 10.1007/s00068-024-02666-w PMC11666754 39251438

[6] Nahm NJ, Como JJ, Wilber JH, Vallier HA. Early appropriate care: definitive stabilization of femoral fractures within 24 hours of injury is safe in most patients with multiple injuries. J Trauma Inj Infect Crit Care. 2011;71(1):175–85. 10.1097/TA.0b013e3181fc93a2. 10.1097/TA.0b013e3181fc93a2 21336198

[7] Pape H-C, et al. The definition of polytrauma revisited: An international consensus process and proposal of the new 'Berlin definition'. J Trauma Acute Care Surg. 2024;77(5):780–6. 10.1097/TA.0000000000000453. 10.1097/TA.0000000000000453 25494433

[8] Lefering R, Huber-Wagner S, Nienaber U, Maegele M, Bouillon B. Update of the trauma risk adjustment model of the Traumaregister DGU™: the revised injury severity classification, version II. Crit Care. 2014;18(5): 476. 10.1186/s13054-014-0476-2. 25394596 10.1186/s13054-014-0476-2 PMC4177428

[9] Pape H-C, Giannoudis PV, Grimme K, Van Griensven M, Krettek C. Effects of intramedullary femoral fracture fixation: what is the impact of experimental studies in regards to the clinical knowledge? Shock. 2002;18(4):291–300. 10.1097/00024382-200210000-00001. 12392270 10.1097/00024382-200210000-00001

[10] Rojer LA, et al. Identifying the severely injured benefitting from a specific level of trauma care in an inclusive network: A multicentre retrospective study. Injury. 2024;55(2):111208. 10.1016/j.injury.2023.111208. 38000291 10.1016/j.injury.2023.111208

[11] Fitschen-Oestern S, et al. Does the time of the day affect multiple trauma care in hospitals? A retrospective analysis of data from the TraumaRegister DGU®. BMC Emerg. 2021;21(1):134. 10.1186/s12873-021-00525-0. 10.1186/s12873-021-00525-0 PMC8590232 34773984

[12] Tan JH, et al. Definitive Surgery Is Safe in Borderline Patients Who Respond to Resuscitation. J Orthop Traum. 2021;35(7):e234–40. 10.1097/BOT.0000000000001999. 10.1097/BOT.0000000000001999 33252447

[13] Imach S, et al. The impact of prehospital tranexamic acid on mortality and transfusion requirements: match-pair analysis from the nationwide German TraumaRegister DGU^®^. Crit Care. 2021;25(1):277. 10.1186/s13054-021-03701-7. 34348782 10.1186/s13054-021-03701-7 PMC8336395

[14] Liu X-Y, Jiang M, Yi C-L, Bai X-J, Hak DJ. Early intramedullary nailing for femoral fractures in patients with severe thoracic trauma: a systemic review and meta-analysis. Chin J Traumatol. 2016;19(3):160–3. 10.1016/j.cjtee.2016.04.001. 27321297 10.1016/j.cjtee.2016.04.001 PMC4908231

[15] Recknagel S. Conversion from external fixator to intramedullary nail causes a second hit and impairs fracture healing in a severe trauma model. J Orthop Res. 2013;31(3):465–71. 10.1002/jor.22242. 23070742 10.1002/jor.22242

[16] Kuhmola A, Simons T, Handolin L, Brinck T. Surgical strategy for femoral shaft fractures in severely injured patients: A 13-year experience from a tertiary trauma centre. Injury. 2021;52(4):956–60. 10.1016/j.injury.2021.01.029. 33541685 10.1016/j.injury.2021.01.029

[17] Weninger P, Figl M, Spitaler R, Mauritz W, Hertz H. Early unreamed intramedullary nailing of femoral fractures is safe in patients with severe thoracic trauma. J Trauma Inj Infect Crit Care. 2007;62(3):692–6. 10.1097/01.ta.0000243203.38466.e0. 10.1097/01.ta.0000243203.38466.e0 17414349

[18] Handolin L, Pajarinen J, Lassus J, Tulikoura I. Early intramedullary nailing of lower extremity fracture and respiratory function in polytraumatized patients with a chest injury: a retrospective study of 61 patients. Acta Orthop Scand. 2004;75(4):477–80. 10.1080/00016470410001277-1. 15370594 10.1080/00016470410001277-1

[19] Dunham CM, et al. Practice management guidelines for the optimal timing of long-bone fracture stabilization in polytrauma patients: the EAST practice management guidelines work group. J Trauma. 2001;50(5):958–67. 10.1097/00005373-200105000-00037. 11379595 10.1097/00005373-200105000-00037

[20] Pfeifer R, et al. Early major fracture care in polytrauma-priorities in the context of concomitant injuries: A Delphi consensus process and systematic review. J Trauma Acute Care Surg. 2024;97(4):639–50. 10.1097/TA.0000000000004428. 39085995 10.1097/TA.0000000000004428 PMC11446538

